# Imaging findings and outcomes after traumatic cerebellar injury: a canine case report

**DOI:** 10.1186/s12917-022-03220-9

**Published:** 2022-03-31

**Authors:** Masamichi YAMASHITA, Yusuke MURAHATA, Inoru YOKOE, Yoshiharu OKAMOTO, Tomohiro IMAGAWA

**Affiliations:** Joint Department of Veterinary Medicine, Faculty of Agriculture, Tottori University, 4-101 Koyama-cho Minami, Tottori, Tottori 680-8553 Japan

**Keywords:** Canine, Traumatic brain injury, Cerebellum, MRI, DWI

## Abstract

**Background:**

Traumatic brain injury (TBI) is a structural injury or physiological disruption of the brain induced by an external force. The cerebellum facilitates movement coordination and provides a sense of equilibrium; damage to this structure can cause a wide variety of symptoms, including ataxia or dystaxia, ocular motor dysfunction, and disequilibrium. TBIs localised to the cerebellum are rare in dogs, and the prognosis following this type of injury remains unclear.

**Case presentation:**

A 10-year-old female Chihuahua/Dachshund-cross dog weighing 2.8 kg presented after a fall of approximately 1 m the preceding night. The dog exhibited paresis of all limbs and was recumbent with constant extensor rigidity with opisthotonos. The bilateral thoracic limb and right pelvic limb spinal reflexes were exaggerated, while the left pelvic limb spinal reflexes were normal. The menace response was decreased, and vertical nystagmus was observed. Magnetic resonance imaging (MRI) revealed a hyperintense lesion on T2weighted (W) images, fluid-attenuated inversion recovery, and diffusion-weighted imaging (DWI). Mannitol and prednisolone were administered, and the dog recovered. The bilateral pelvic limb postural reactions improved by Day 16. On Day 22, MRI revealed a decrease in the hyperintense area of the T2W images, and this lesion appeared isointense on DWI.

**Conclusions:**

In this case report, a dog with localised injury to the cerebellum that comprised a post-tentorial lesion recovered with a favourable outcome. Moreover, similar to reports in humans, DWI can help diagnose and evaluate TBI in dogs.

## Background

Traumatic brain injury (TBI) is caused by a sudden impact to the head an external force, resulting in a structural injury or physiological disruption [1, 2]. The modified Glasgow Coma Scale (MGCS) is the grading system of preference for the evaluation of patients affected by TBI in veterinary medicine. It evaluates three major categories: motor activity, brainstem reflexes, and level of consciousness [2, 3]. TBI can also be graded using magnetic resonance imaging (MRI), which is becoming more common with the increase in the accessibility of the MRI equipment. Certain MRI findings, including non haemorrhagic contusions, brainstem lesions, and diffuse axonal injury, are more sensitive predictors of clinical outcomes [2, 4].

The cerebellum facilitates the coordination of movement and provides a sense of equilibrium [5]. The cerebellum is also thought to coordinate several external senses, motion itself, and even higher cognitive function [5], although the mechanisms underlying these processes are not entirely clear. Damage to the cerebellum can cause a wide variety of symptoms, including ataxia or dystaxia, ocular motor dysfunction, and disequilibrium [5, 6].

Tumours and cerebrovascular disease are the most common causes of cerebellar disorder in dogs [6]. Traumatic injury to the cerebellum can also occur, although these injuries are generally accompanied by damage to the cerebrum and/or brainstem. TBIs localised to the cerebellum are rare in dogs, and the prognosis following this type of injury remains unclear. In humans, traumatic injury to the cerebellum is mostly accompanied by damage to the brainstem, and the prognosis in these cases is poor, likely because of the differences in the anatomical characteristics of humans and dogs [7].

In this case report, we reported the imaging findings and outcomes in a dog following a TBI localised to the cerebellum.

## Case presentation

A 10-year-old female Chihuahua/Dachshund-cross dog weighing 2.8 kg presented after a fall of approximately 1 m the preceding night. Seventeen hours after the accident, the dog presented in lateral recumbency; there was opisthotonus with thoracic limb extension (decerebellate rigidity). There was non-ambulatory tetraparesis. The mental status was normal. The dog was anorexic since the accident and unable to drink unaided. After the injury, the dog’s neck was stabilised with a neck collar.

The neurological examination revealed loss of postural reactions in all the limbs. The bilateral thoracic limb and right pelvic limb spinal reflexes were exaggerated, while the left pelvic limb spinal reflexes were normal. The menace response was bilaterally decreased, and vertical spontaneous nystagmus was present. The dog’s MGCS score was 14.

**Fig. 1:**
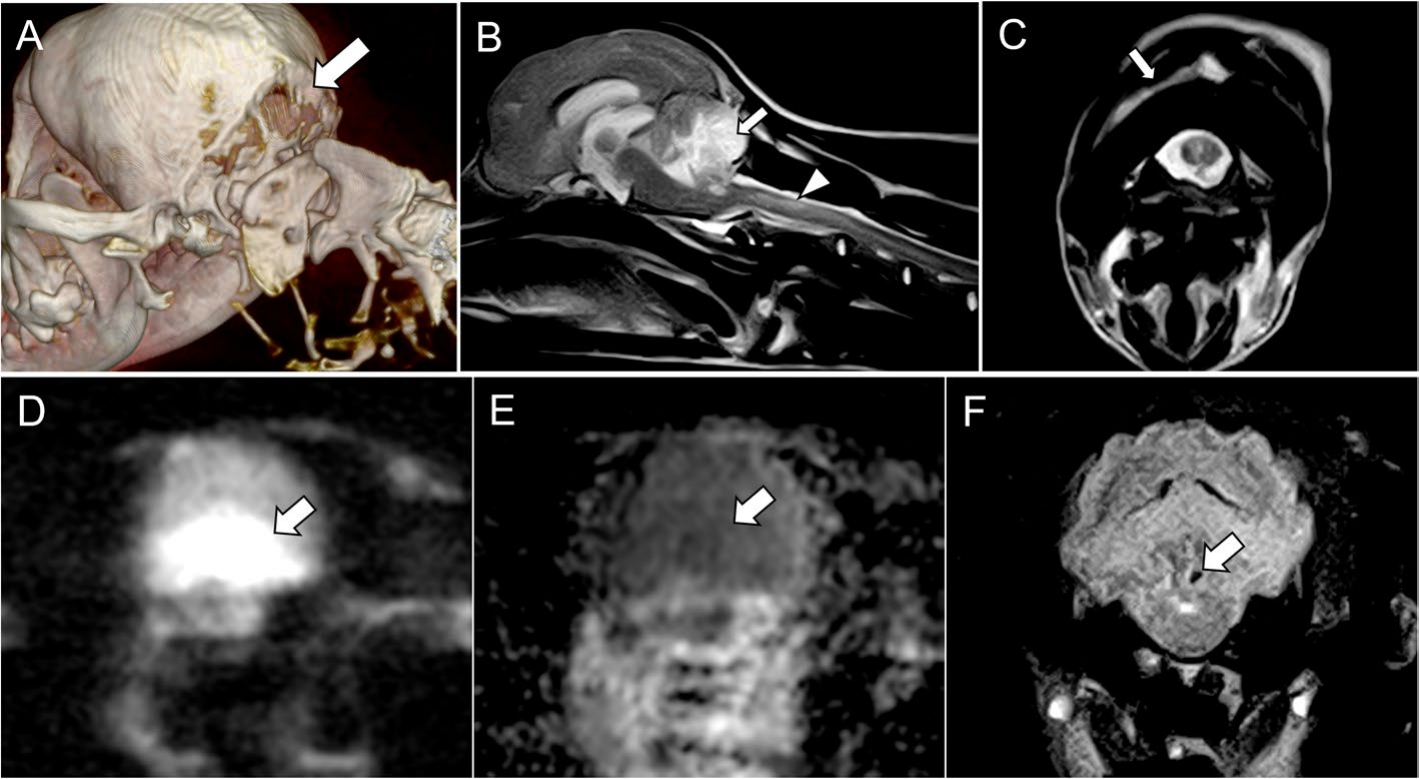
Imaging findings on Day 1 (17 h after traumatic brain injury). (A) Three-dimensional computed tomography shows incomplete ossification of the supraoccipital bone (white arrow). Vertical extension of the foramen magnum is observed. (B) T2-weighted magnetic resonance imaging (MRI) shows a hyperintense lesion in the caudal half of the cerebellum (white arrow) and a hyperintense lesion in the spinal cord extending for all lengths of C2 vertebral body (white arrowhead). Hyperintense in olfactory build and ventral aspect of the frontal lobe is partial volume artifact. (C) T1-weighted MRI shows a hyperintense lesion between the rectus capitis dorsalis major and the obliquus capitis caudalis muscles. (D) Diffusion-weighted imaging (DWI) shows a hyperintense cerebellar lesion (white arrow). (E) Apparent diffusion coefficient (ADC) mapping shows a hypointense cerebellar lesion (white arrow). (F) T2*-gradient-recalled echo imaging shows a small, hypointense lesion within the cerebellar lesion (white arrow)

A complete blood cell count and serum biochemical analysis revealed a mild reduction in the red blood cells and haematocrit, a moderate elevation in creatine phosphokinase and glucose levels, and an increase in the C-reactive protein levels. Computed tomography (CT) was performed using a 16-slice CT scanner (ECLOS, HITACHI, Ltd., Tokyo, Japan). The images were acquired in the transverse plane and reconstructed with a slice thickness of 2.5 mm with a bone and a soft tissue algorithm. The dose parameters were 100 kV and 175 mA. CT examination revealed incomplete ossification of the supraoccipital bone (Fig. 1A) and did not reveal bone fracture or haematoma in the brain. MRI (0.3-T AIRIS Vento, HITACHI, Ltd.; Tokyo, Japan) revealed lesions in the spinal cord, cerebellum, and the paraspinal muscles at the level of the neck (Fig. 1B). T2 weighted (W) images depicted a hyperintense lesion in the spinal cord at the level of the second cervical vertebra (C2) and dilation of the central canal of the spinal cord from C3 to C5 (Fig. 1B). T2W images of the cerebellum revealed areas of hyperintensity in the caudal vermis, cerebellar hemispheres, and flocculus, and fluid-attenuated inversion recovery (FLAIR) imaging also revealed hyperintensities in the same areas. The lesions remained hyperintense on FLAIR, compatible with pre-syrinx/ syrinx formation, oedema, and/or gliosis. The lesions appeared hyperintense on diffusion-weighted imaging (DWI) (b value = 1000 s/mm^2^) and isointense or hypointense on apparent diffusion coefficient (ADC) mapping (0.80 × 10^− 3^ mm^2^/sec) (Fig. 1D, E). Moreover, the lesion appeared isointense on T1W images. A small hypointense lesion within the cerebellar lesion was visible on T2*-gradient-recalled echo (GRE) imaging (Fig. 1F). Moreover, there was generalised sulcal effacement at the level of the cerebrum and cerebellum; the fourth ventricle was subjectively decreased in size, leading to indirect signs of brain oedema and increased intracranial pressure. There were hyperintense T2W lesions at the level of the paraspinal muscles of the neck between the rectus capitis dorsalis major muscle and obliquus capitis caudalis muscle (Fig. 1C). The sulcus of the cerebrum in general was unclear, and the third ventricle was enlarged. Any detectable lesions were not observed in the cerebrum or brainstem.

After the MRI examination, mannitol 2 g/kg (Yoshindo Inc., Toyama, Japan) was administered intravenously for 30 min, and prednisolone 1 mg/kg/daily (Kyoritsu Seiyaku Corporation, Tokyo, Japan) was injected subcutaneously for 2 days. The dog received syringe feeding by Day 2 and could eat independently by Day 3. Prednisolone 1 mg/kg (Teva Takeda Yakuhin Ltd., Nagoya, Japan) was subsequently prescribed for 1 week. By Day 8, the dog’s appetite had improved, the opisthotonos had decreased, and the dog could walk a short distance. Moreover, the right thoracic limb postural reaction improved, and the vertical nystagmus disappeared. The bilateral pelvic limb postural reactions improved by Day 16. On Day 22, follow-up was performed by MRI.

**Fig. 2:**
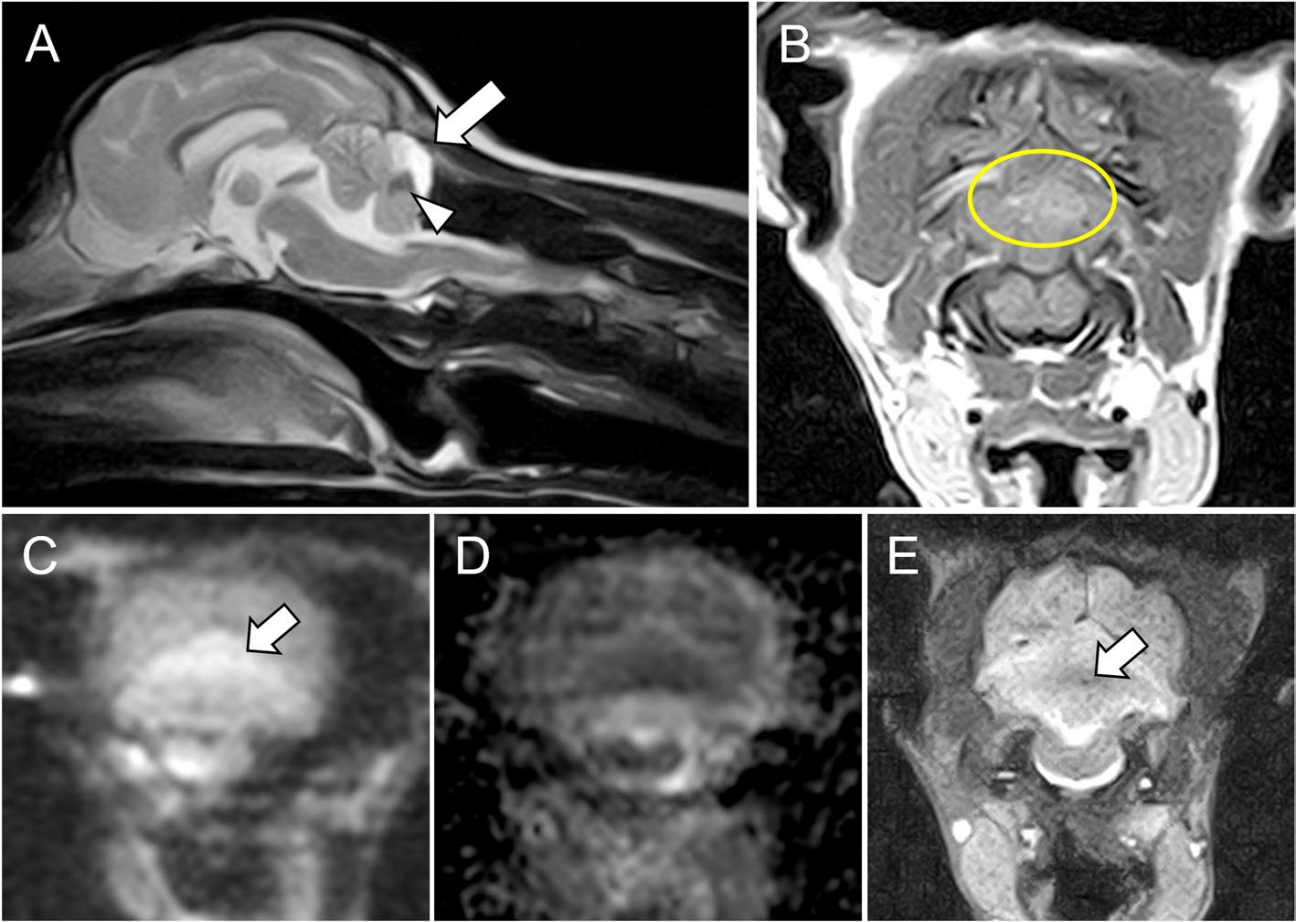
Magnetic resonance imaging (MRI) performed 21 days after traumatic brain injury. (A) MRI shows a decrease in the area of the hyperintense lesion (white arrow), with a portion of this lesion becoming hypointense (white arrowhead). (B) T1-weighted MRI shows a hyperintense lesion with weak enhancement after intravenous administration of a paramagnetic contrast agent (yellow circle). (C) In diffusion-weighted imaging (DWI), the signal that the hyperintense lesion in Day 1 is decreasing and approaching isointense compared to in Day 1. (D) Apparent diffusion coefficient (ADC) mapping reveals a hypointense cerebellar lesion, and the intensity did not change (white arrow). (E) The hypointense lesion became smaller in the T2*-gradient-recalled echo imaging, and the location of the lesion was consisted the location of the with hypointense lesion in T2WI (white arrow). By T2WI and T2*, the signal of the lesion initiated intracellular hemosiderin

On Day 22, MRI revealed that the area and intensity of the cerebellum lesion decrease in T2W images, and a portion of this lesion became hypointense (Fig. 2A). This lesion showed showing weak enhancement on T1W imaging following intravenous administration of gadopentetate dimeglumine (Magnevist, Bayer Yakuhin Ltd., Osaka, Japan) (Fig. 2B). The intensity of this lesion had decreased on DWI and was hypointense or near isointense on ADC mapping (0.98 × 10^− 3^ mm^2^/sec), while the small lesion identified on T2*-GRE imaging had decreased (Fig. 2C, D). The size of the T2W images hyperintense lesion in C2 in the T2W images had decreased, and expansion of the central canal was no longer observed. The volume of the cerebellum was reduced, and the size of the fourth ventricle was increased (Fig. 2A). The T2W images lesion in the fascia of the rectus capitis dorsalis major in the T2W images had disappeared. At this point, the dog was ambulatory without assistance, exhibited mild tetraparesis and was able to lead a good quality of life.

## Discussion and conclusion

TBIs to the cerebellum have been described in previous studies using MGCS; however, it is rare for the cerebellum to be damaged without concomitant injury to the brainstem and cerebrum. The outcomes of injuries localised to the cerebellum have never been reported in dogs [2, 6]. In the present case, central nervous systems symptoms, such as nystagmus, which may have been caused by cerebral oedema were also present, brain damage was mostly localised to the cerebellar region, and strong cerebellar symptoms were evident. Despite this injury, the dog showed symptomatic improvement and treatment yielded a good outcome. Moreover, this study presented pertinent findings on DWI and ADC mapping in a dog with TBI, which have not been previously reported.

In the present case, MRI examinations were conducted 17 h and 22 days after the accident. At 17 h, the cerebellar lesion was clearly hyperintense on the T2W images and FLAIR, and a microhaemorrhage was detected on the T2*-GRE. T2W images, FLAIR, and T2*-GRE are important imaging sequences for evaluating TBI [2], with the localisation and volume of lesions related to the prognosis of TBI in humans, and these sequences were important for evaluating TBI in this case. We believe that the small volume of bleeding and minimal damage to the brainstem were responsible for the patient’s good outcome. In dogs, DWI and ADC mapping have been used in previous reports on cerebrovascular disease [8, 9]. The findings of hyperintense DWI and hypointense ADC mapping in this case were similar to the reported findings for cerebrovascular disease in dogs. The point of differentiation between cerebrovascular disease and TBI in the present case is the trauma in the soft tissue around the occipital area. If the cause of the nervous system symptoms is not clear, imaging of the soft tissue around the head may help in diagnosis.

In this case, the lesion was also evident on DWI and ADC mapping 17 h and 22 days following the accident. DWI and ADC mapping reflect changes in the diffusion of water molecules in the brain tissue, and restricted diffusion is observed in areas with cell death due to significant tissue injury. Therefore, DWI and ADC mapping are MRI sequences with sensitivity for detecting ischaemic injury [10, 11]. DWI and ADC mapping can detect cytotoxic oedema by measuring the random motion of water protons, a process that is reduced following cellular injury attributed to a variety of causes [10, 11]. In human medicine, the findings of quantitative DWI and ADC mapping are related to prognosis following TBI and stroke, and a high percentage of hyperintense area of DWI and hypointense area of ADC in the brain indicates a poor prognosis [10, 11].. In the present case, the cerebellar lesion appeared hyperintense on DWI and hypointense on ADC mapping 17 h after the accident. However, these intensities transformed to near isointensity by 22 days after the accident, possibly reflecting the improvements in oedema.

In the present case, the MGCS score was 14, and the MRI grade was II [2, 3, 12]. The dog exhibited evidence of cerebellar damage with cerebellar symptoms. In accordance with both MGCS and the MRI grading, the outcome had been favourable. Recent reports indicate that CT is limited in predicting short-term prognosis [13]. In canine TBI without obvious bleeding, haematoma, or fracture, as in the present case, CT may not contribute to the diagnosis. On MRI, the lesion included the caudal half of the vermis, the cerebellar hemispheres, and the flocculus. In cerebellar injuries, a strong sense of disequilibrium is considered to result from damage to the caudal vermis, especially the flocculonodular lobe, while ocular motor dysfunction is thought to result from damage to the flocculus [14]. Dysmetria, which is caused by damage to the cerebellar hemispheres and part of the vermis such as the declive, folium, tuber of the vermis, simplex lobule and ansiform lobule, was not observed in this case, probably due to a strong sense of disequilibrium [5, 14]. Twenty-two days after the accident, the structure of the caudal cerebellum had disappeared or atrophied and was instead filled with cerebral fluid; the symptoms had improved, possibly due to the high plasticity of the cerebellum [15].

TBI treatment frequently involves corticosteroid and mannitol administration with the goal of alleviating brain oedema and signs of increased intracranial pressure within the brainstem, as well as to present brain herniation [6]. In the present case, it was considered that the administration of corticosteroid and mannitol prevented enlargement of oedema and inflammation in the cerebellum.

In this case report, a dog with localised injury to the cerebellum that comprised a post-tentorial lesion recovered with a favourable outcome. Moreover, quantitative DWI and ADC mapping provided useful information to aid in diagnosis and evaluation of brain MRI for a dog affected by TBI; these could be valuable prognostic aids in the future as they are in human neuroradiology.

## Data Availability

The data are not available for public access because of patient privacy concerns, but are available from the corresponding author on reasonable request.

## Abbreviations

TBI: Traumatic brain injury
MRI: Magnetic resonance imaging
W: Weighted
DWI: Diffusion-weighted imaging
MGCS: Modified Glasgow Coma Scale
CT: Computed tomography
C2: The second cervical vertebra
FLAIR: Fluid-attenuated inversion recovery
ADC: Apparent diffusion coefficient
GRE: Gradient-recalled echo

## References

[1] Management of Concussion/mTBI working group.VA/DoD clinical practice guideline for Management of Concussion/mild traumatic brain injury. J Rehabil Res Dev. 2009;46:1–68.

[2] Beltran E, Platt SR, McConnell JF, Dennis R, Keys DK, De Risio L (2014). Prognostic value of early magnetic resonance imaging in dogs after traumatic brain injury: 50 cases. J Vet Intern Med.

[3] Platt SR, Radaelli ST, McDonnell JJ (2001). The prognostic value of the modified Glasgow coma scale in head trauma in dogs. J Vet Intern Med.

[4] Bastian AJ, Lisberger SG, Kandel ER, Koester JD, Mack SH, Siegelbaum SA (2013). The cerebellum. Principles of neural science.

[5] de Lahunta A, Glass EN, Kent M. Cerebellum. Lahunta’s veterinary neuroanatomy and clinical neurology. Philadelphia: Elsevier. 2021:374–413.

[6] Cizinauskas S, Jaggy A, Jaggy A, Couteur RL (2010). Cerebellum. Atlas and textbook of small animal neurology: an illustrated text.

[7] Nakamura N, Owada S, Sekino H, Sakai H (1976). Traumatic cerebellar dysfunction. Neurol Med Chir.

[8] McConnell JF, Garosi L, Platt SR (2005). Magnetic resonance imaging findings of presumed cerebellar cerebrovascular accident in twelve dogs. Vet Radiol Ultrasound..

[9] Sutherland-Smith J, King R, Faissler D, Ruthazer R, Sato A (2011). Magnetic resonance imaging apparent diffusion coefficients for histologically confirmed intracranial lesions in dogs. Vet Radiol Ultrasound.

[10] Shakir A, Aksoy D, Mlynash M, Harris OA, Albers GW, Hirsch KG (2016). Prognostic value of quantitative diffusion-weighted MRI in patients with traumatic brain injury. J Neuroimaging.

[11] Thomas RGR, Lymer GK, Armitage PA, Chappell FM, Carpenter T, Karaszewski B (2013). Apparent diffusion coefficient thresholds and diffusion lesion volume in acute stroke. J Stroke Cerebrovasc Dis.

[12] Lagares A, Ramos A, Pérez-Nuñez A, Ballenilla F, Alday R, Gómez PA (2009). The role of MR imaging in assessing prognosis after severe and moderate head injury. Acta Neurochir.

[13] Wyatt S, Llabres-Diaz F, Lee CY, Beltran E (2021). Early CT in dogs following traumatic brain injury has limited value in predicting short-term prognosis. Vet Radiol Ultrasound..

[14] Brazis PW, Brazis PW, Masdeu JC, Biller J (2007). The cerebellum. Localization in clinical neurology.

[15] Jörntell H, Ekerot C (2002). Reciprocal bidirectional plasticity of parallel fiber receptive fields in cerebellar Purkinje cells and their afferent interneurons. Neuron.

